# Supplementary material for: Magnetic Properties Controlled by Interstitial or Interlayer Cations in Iron Chalcogenides
Source: Sci Rep. 2016 Jan 8;6:19031. doi: 10.1038/srep19031 (PMC4705632; doi:10.1038/srep19031)
Supplement: Supplementary Information [file srep19031-s1.pdf]

Scientific Reports

nature publishing group npg

OPEN ACCESS Article Processing Charge (APC) Payment Form

Scientific Reports is an open access journal, where all journal content is permanently available free of charge to all readers. Articles are therefore subject to an article processing charge in order to support publication costs (as detailed in the 'Instructions to Authors').

It is mandatory to send this completed form and the Open Access License to Publish form to the return address (details below). Your manuscript may not be processed for publication until we have received the relevant forms.

Credit terms are 30 days from receipt of invoice. Failure to pay your invoice within the stated credit term may make you liable to such penalties as restrictions on your ability to publish with NPG or this title in the future, involvement of a third party debt collection agency and legal proceedings.

The APC will be charged at RMB 9,900

VAT will be added where applicable.
China: payment is received on behalf of Nature Publishing Group (NPG) by Beijing ZhongKe I/E Company (BJZK) / 此声明兹证明Nature Publishing Group (简称 NPG) 授权北京中科进出口有限责任公司 (简称中科公司) 为 Nature 出版社在中华人民共和国大陆地区版面費独家代理商

Manuscript Details (COMPLETE IN BLOCK CAPITALS)

Title of article:
Magnetic Properties Controlled by Interstitial or Interlayer Cations in Iron Chalcogenides

Manuscript number:
SREP-15-19562A

Corresponding author name:
Yu-Zhong Zhang

Corresponding author email address:
yzzhang@tongji.edu.cn

Billing Details (COMPLETE IN BLOCK CAPITALS)

Contact name:
Yu-Zhong Zhang

Organization name:
Tongji University

Billing address:
School of Physics Science and Engineering,
Tongji University,
Siping Rd. 1239#,
Shanghai 200092, China

Email Address:
yzzhang@tongji.edu.cn

Tel Number:
(Mobile number for mainland Chinese customers / 请在中国大陆的客户输入您的手机号码)
18019787157

Postcode/ZIP:
200092

Payment Method:
☑ **Invoice** *An invoice will be sent by post and email.*
Payment is required within 30 days.
PO Number (if required):

VAT / GST / Tax Number:

TAX INFORMATION

CHINA - Please note that VAT will be added at the mainland China standard rate if the invoice is issued to individuals, primary and secondary schools, international schools, public libraries, corporations or government organizations: 中国大陆客户: 如果您需要开具的发票单位为个人、中小学校、国际学校、公共图书馆、公司、政府组织等, 根据中国税务规定, 在出版费之外, 支付相应的增值税
中国大陆客户: 请提供中国大陆手机号码

PLEASE RETURN YOUR COMPLETED PAYMENT FORM AND OPEN ACCESS LICENSE TO PUBLISH FORM TO:
Scientific Reports **Email:** scientificreports@nature.com **Fax:** +44 (0)20-7843-4811
